# Vascular Dysfunction in Alzheimer’s Disease: Alterations in the Plasma Contact and Fibrinolytic Systems

**DOI:** 10.3390/ijms24087046

**Published:** 2023-04-11

**Authors:** Ana Badimon, Daniel Torrente, Erin H. Norris

**Affiliations:** Patricia and John Rosenwald Laboratory of Neurobiology and Genetics, The Rockefeller University, 1230 York Avenue, New York, NY 10065, USA

**Keywords:** Alzheimer’s disease, contact system, vasculature, fibrinogen, beta-amyloid

## Abstract

Alzheimer’s disease (AD) is the most common neurodegenerative disease, affecting millions of people worldwide. The classical hallmarks of AD include extracellular beta-amyloid (Aβ) plaques and neurofibrillary tau tangles, although they are often accompanied by various vascular defects. These changes include damage to the vasculature, a decrease in cerebral blood flow, and accumulation of Aβ along vessels, among others. Vascular dysfunction begins early in disease pathogenesis and may contribute to disease progression and cognitive dysfunction. In addition, patients with AD exhibit alterations in the plasma contact system and the fibrinolytic system, two pathways in the blood that regulate clotting and inflammation. Here, we explain the clinical manifestations of vascular deficits in AD. Further, we describe how changes in plasma contact activation and the fibrinolytic system may contribute to vascular dysfunction, inflammation, coagulation, and cognitive impairment in AD. Given this evidence, we propose novel therapies that may, alone or in combination, ameliorate AD progression in patients.

## 1. Introduction

The World Health Organization estimates that over 55 million people worldwide have dementia and expects this number to rise to 139 million by 2050 [1]. Alzheimer’s disease (AD) is the most common form of dementia, making up 60–80% of cases worldwide [1].

Pathological hallmarks of the AD brain include the accumulation of the beta-amyloid peptide (Aβ) and hyperphosphorylated tau, which are present in the form of plaques and neurofibrillary tangles, respectively [2]. These proteinaceous deposits are collectively thought to drive the inflammation, degeneration, and subsequent cognitive deficits observed in patients. Over 80% of AD patients also exhibit cerebral amyloid angiopathy (CAA), which is the deposition of Aβ in and around blood vessels of the brain [3].

Toxic Aβ is generated from the cleavage of the amyloid precursor protein (APP) by γ-secretase. Genetic studies provide compelling evidence that Aβ is a key player in AD pathogenesis. For example, autosomal dominant AD is caused by mutations that influence Aβ production and/or toxicity. Autosomal dominant AD mutations are all found within three genes: PSEN1 and PSEN2, whose proteins, presenilin-1 and -2, form the catalytic subunit of γ-secretase, or APP itself [2]. Mutations in APP have been identified both within the Aβ sequence (amino acids 672–718), which may cause changes to the structure and binding properties of Aβ, or in upstream/downstream regions of APP, which may lead to changes in Aβ cleavage and production [2]. To date, researchers have identified 98 APP mutations and over 400 PSEN1/2 mutations that cause autosomal dominant AD (AlzForum). 

Interestingly, familial AD patients make up only 1–5% of cases [2,4]. The majority of AD cases are sporadic and present in patients older than 65 years of age. Genetic mutations in APOE (apolipoprotein E) [5], MAPT (tau) [6], TREM2 (triggering receptor expressed on myeloid cells) [7,8], MS4A4(membrane-spanning 4-domains A4) [9,10], CD33 [9,10], ABCA7(ATP binding cassette subfamily 7) [9], and CLU (clusterin) [11] have been linked to AD via genome-wide association studies (GWAS) or by pathological examination (AlzForum). These mutations do not run in families and are therefore considered sporadic in nature. The identification of non-Aβ/APP risk factors has widened the scope of alternative factors that may drive AD pathogenesis, such as neuroinflammation and vascular dysfunction. A recent study in AD patients and non-demented individuals found that 30 of the top 45 GWAS-identified risk genes are enriched in vascular cells [12]. In support of this finding, longitudinal studies have found that vascular pathology precedes a cognitive decline in both familial and sporadic AD [13,14].

Importantly, vascular dysfunction alone—in the absence of Aβ plaques or tau tangles—can lead to neurodegeneration and cognitive dysfunction. This condition, referred to as vascular dementia, is the second most common type of dementia after AD [15]. In vascular dementia, the blood-brain barrier, which typically protects the brain from pathogens, regulates cerebral blood flow and controls the transport of oxygen and nutrients into the brain, is damaged or dysfunctional. The underlying cause of vascular dementia can be varied among patients [16]. Often, patients present with a stroke, or a history of a stroke followed by cognitive deficits [16]. Of note, patients can be diagnosed with vascular dementia, AD, or both, depending on the clinical presentation and pathological findings. 

More recently, the focus on vascular factors that are involved in AD has brought to light other physiological systems that may be altered in this disease. These pathways include the plasma contact system and the fibrinolytic system. The plasma contact system is a protease cascade in the circulation that, when activated, promotes coagulation, inflammation, and vascular leakage. The fibrinolytic system is necessary for the degradation of blood clots. Dysfunction of both pathways has been linked to AD [17], which may explain some of the clinical manifestations of vascular dysfunction observed in AD patients.

## 2. Clinical Manifestations

A major hallmark of AD is the presence of proteinaceous inclusions of Aβ and tau in the brain. However, the accumulation of these proteins in the brain does not guarantee dementia. This discrepancy, along with the modest cognitive rescue achieved by recently approved anti-amyloid antibody treatments [18,19,20], suggests that other systems may be involved in driving disease pathology, including inflammation and vascular dysfunction [17].

Longitudinal studies have shown that vascular deficiencies, including changes in blood-brain barrier integrity, brain microbleeds, white matter hyperintensities, small vessel disease, and changes in resting cerebral blood flow, precede cognitive dysfunction in AD patients [14]. In fact, one of the earliest detectable vascular changes is the reduction in cerebral blood flow. AD patients show a 10–20% reduction in blood flow prior to the onset of dementia [21,22]. Expected age-related structural changes in blood vessels, such as increased vessel wall thickness, decreased vessel wall elasticity, and increased vessel wall resistance, are exacerbated in AD patients [21]. This combination likely contributes to hypoperfusion and cerebrovascular dysregulation. Lower cerebral blood flow correlates with worse cognitive impairment [21]. In line with this finding, a sudden but temporary decrease in cerebral blood flow is sufficient to induce cognitive deficits and affect performance in sustained attention tasks [23]. In contrast, patients over 55 years of age with higher cerebral blood flow were significantly less likely to be diagnosed with dementia within 6.5 years [24]. Typically, cerebral blood flow increases locally in response to enhanced neuronal activity, a phenomenon referred to as neurovascular coupling [25]. Inadequate blood flow resulting from deficits in neurovascular coupling creates an imbalance in the energy supply and demand for active neurons, which may, in turn, drive deficits in cognitive function. This imbalance, along with other alterations in the vasculature, diminishes the supply of oxygen, metabolites, and nutrients to the brain [26]. 

Vascular damage induced by altered cerebral blood flow is further exacerbated by impairment of the blood-brain barrier in AD. Recent studies have found that the structural breakdown of the blood-brain barrier is due in part to pericyte degeneration [12]. Pericytes fortify the blood-brain barrier by surrounding endothelial cells [27]. Imaging studies have elucidated that the hippocampus is particularly vulnerable to pericyte-dependent blood-brain barrier breakdown and that this damage worsens with age [28]. Single-nuclei RNA-sequencing of human vascular cells (VINE-seq, Vessel Isolation and Nuclei Extraction for Sequencing) identified numerous changes in the vasculature of AD patients compared with non-demented control individuals [12]. The study identified an overall decrease in recovered nuclei in AD patients, suggesting a loss in vascular cells, which was confirmed by immunostaining. Moreover, gene expression changes were revealed in mural cells and fibroblasts, cells critical in maintaining blood-brain barrier structure, suggesting dysregulated vasoconstriction and compromised blood flow [12]. These alterations may underlie the functional changes in cerebral blood flow detected in patients with AD. 

Results from GWAS studies have driven researchers to focus heavily on the contribution of immune cells to AD pathology. However, novel techniques like VINE-seq that allow for accurate profiling of vascular cells have shed light on how genetic variants may drive vascular changes as well. GWAS studies have identified dozens of AD risk genes that are primarily expressed in vascular cells [12], suggesting that the integrity of vascular and perivascular cells is important in AD. Of note, blood-brain barrier breakdown has been linked to cognitive dysfunction even in the absence of Aβ and tau deposition, as seen in vascular dementia [29]. Thus, loss of vascular integrity may be sufficient to drive dementia. 

Impaired vasculature allows for the interaction of blood and brain proteins, which can be detrimental. A strong example of crosstalk between AD pathology and vascular dysfunction is cerebral amyloid angiopathy (CAA) [3,30], a condition in which Aβ deposits in and around the blood vessels of the brain. An estimated 80–95% of patients with AD develop CAA. The accumulation of Aβ results in endothelial damage, loss of vascular integrity, and inflammation. Consequently, patients with CAA have a higher incidence of infarcts compared to those without CAA [30]. Furthermore, vascular damage resulting from CAA likely drives neurological changes, as hereditary forms of CAA are associated with decreased functional brain connectivity [31]. 

The presence of CAA facilitates the interaction of Aβ with blood proteins, such as fibrinogen, as the vascular damage caused by CAA promotes vascular leakage. Fibrinogen is integral in clot formation. The binding of Aβ and fibrinogen causes perturbations in clot structure and delayed fibrinolysis [32]. Certain mutations in Aβ that have been linked to early onset AD result in strong binding affinities between Aβ and fibrinogen, exacerbating deposition along the vasculature and increasing vascular dysfunction [33]. 

Compounding the effects of vascular dysfunction in AD is the contributing role of neuroinflammation [34,35]. Postmortem studies have repeatedly found evidence of increased inflammation in AD patients’ brains [36]. Longitudinal imaging studies have found that AD patients exhibit chronic inflammation throughout the disease duration [37]. In animal model systems, inhibiting inflammation and preventing microglial activation can improve AD pathology and cognition [38,39]. Aβ plaques can directly activate microglia, the main immune cells in the brain, and astrocytes [35]. Activated microglia release a variety of proinflammatory and toxic products, including cytokines, reactive oxygen species, and nitric oxide [36]. The presence of Aβ and increased production of cytokines can further damage other cells in the brain, such as neurons, thereby promoting neurodegeneration. Recent GWAS studies have strongly implicated microglia in AD progression, as their role in immune function and in phagocytosis is particularly important in AD pathogenesis [40]. In addition, chronic inflammation can lead to endothelial cell dysfunction, which results in vascular damage and leakage [41]. Inflammation-driven vascular damage may play a role in slowing cerebral blood flow and allowing blood proteins to extravasate into the brain, leading to eventual cognitive dysfunction. However, the mechanisms by which vascular abnormalities and inflammation are initiated in AD and how they might affect neurons remain unclear.

## 3. The Plasma Contact System

The plasma contact system is a protease cascade in the blood. When activated, it promotes coagulation and inflammation. The plasma contact system is initiated by the activation of coagulation factor XII (FXII) by negatively charged surfaces, such as Aβ. The prothrombotic branch of this system is driven by FXII-mediated activation of factor XI (FXI), resulting in fibrin clot formation. Aberrant activation of this system can lead to vessel occlusion, microinfarcts, hypoperfusion, and inflammation, all observed in AD. The contact system can also promote inflammation via the activation of prekallikrein (PK), resulting in the cleavage of high molecular weight kininogen (HK) and the release of bradykinin. Bradykinin generation triggers increased vascular permeability and inflammation via activation of the bradykinin 1 and bradykinin 2 receptors. Thus, by promoting both vascular dysfunction and inflammation, aberrant activation of the plasma contact system can promote both these pathologies.

Aβ can bind to and activate FXII to trigger both thrombotic and inflammatory pathways [42,43]. Although the exact mechanism by which Aβ activates the plasma contact system is unknown, there are two main possibilities. In AD patients, Aβ from the brain is transported across the blood-brain barrier into the blood, where it can activate the contact system [44]. In addition, the majority of patients with AD exhibit CAA, which facilitates the direct interaction between Aβ and plasma proteins to promote contact system activation. Vascular damage in AD allows for the extravasation of plasma proteins into the brain. Consequentially, fibrinogen, FXII, and HK have been found in the brains of patients with AD, often co-depositing with Aβ plaques [45,46].

Patients with AD exhibit increased activation of the contact system [47,48,49]. AD patients show reduced levels of intact FXII and increased levels of activated FXII [50]. This finding is linked to increased kallikrein activity and increased cleavage of HK, resulting in reduced intact HK levels and increased cleaved HK and bradykinin levels in plasma [51,52,53,54]. Importantly, the extent of contact system activation was found to correlate with cognitive decline [53,54]. Patients with worse cognitive decline (higher Mini-Mental State Examination or MMSE scores) exhibit significantly higher plasma bradykinin levels [53]. An analysis of mild cognitively impaired (MCI) patients showed that individuals that exhibit impaired memory recall have increased kallikrein activity compared to those patients with intact memory recall [54]. This result suggests that an activated contact system and increased bradykinin production are involved in AD pathogenesis, which could explain at least some of the inflammation observed in AD patients. Bradykinin can also promote edema, vasodilation, and vascular permeability. In addition to exhibiting changes in overall levels of contact system proteins, the enzymatic activity of these factors is increased [47].

Furthermore, AD patients show increased activation of the prothrombotic arm of the contact system. Patient plasma shows decreased FXI, indicating increased activated FXI (FXIa) levels [51]. In line with this finding, AD patients exhibit prolonged activated partial thromboplastin time (aPTT), suggesting abnormal clotting via the intrinsic pathway [55]. Clot initiation and the rate of clot formation are significantly delayed in plasma from AD patients [55]. Consequently, the time to achieve maximum clot strength (Tmax) is also delayed [55]. These abnormalities in FXI-mediated intrinsic clotting correlate to cognition, as patients with more delayed clotting have a lower cognitive function as determined by MMSE [55].

The aforementioned alterations in contact system activation and intrinsic clotting have been mirrored in mouse models. Plasma from AD mice exhibits increased activation of the plasma contact system [42,56] and prolonged aPTT [55]. Murine studies have found that inhibition of the plasma contact system can rescue cognitive function in mice and reduce the level of neuroinflammation associated with AD [56]. In this study, the knockdown of plasma FXII was achieved by administering an antisense oligonucleotide, a short synthetic strand of nucleotides that targets a specific mRNA sequence to modulate and limit protein translation. Administration of an antisense oligonucleotide against FXII in mice knocked down plasma levels of FXII, inhibited the plasma contact system, and resulted in reduced neuroinflammation, less vascular damage, and improved cognitive function in AD mice compared with AD mice treated with a scrambled control antisense oligonucleotide. Collectively, these data highlight the potential of the plasma contact system as a novel therapeutic target.

## 4. The Fibrinolytic System 

The fibrinolytic system plays a crucial role in maintaining vascular integrity and regulating blood clot formation by lysing fibrin clots in both healthy and disease conditions. In addition to hemostasis, the fibrinolytic system also plays crucial roles in wound healing, tissue remodeling, and inflammation [57,58,59]. This system is composed of various proteins, including plasminogen, tissue plasminogen activator (tPA), and urokinase plasminogen activator (uPA). tPA and uPA are serine proteases that can proteolytically activate plasminogen to plasmin, which can then degrade fibrin clots to prevent excess blood clot formation. This system is regulated by plasminogen activator inhibitor-1 (PAI-1). PAI-1 binds to tPA or uPA, preventing their activation of plasminogen to plasmin. 

Abnormalities in the fibrinolytic system have been implicated in a range of diseases, including cardiovascular disease, stroke, and AD [60,61]. In AD, many fibrinolytic system components have been implicated in contributing to the accumulation of Aβ and, to a lesser extent, tau [62,63]. 

### 4.1. Plasminogen 

Plasmin is a serine protease that is generated from plasminogen by the proteolytic activation of tPA or uPA. Plasminogen is expressed and secreted primarily by hepatocytes in the liver [64]. The primary function of plasmin is to lyse fibrin clots, which are formed in response to injury or inflammatory activity. This process is known as fibrinolysis, which is critical for the maintenance of normal blood flow and tissue repair. In addition to its role in blood clot resolution, plasmin is also involved in brain function. In the brain, plasminogen is expressed and secreted by neurons [65], and it has been associated with the regulation of synaptic activity by affecting long-term potentiation through the maturation of brain-derived neurotrophic factor (BDNF) [66]. Plasminogen’s conversion to plasmin has been implicated in a variety of pathological processes in the brain, including neurodegeneration and neuroinflammation [67,68].

In AD, it has been suggested that plasmin aids in the clearance of Aβ, as demonstrated by delayed Aβ clearance in plasminogen-deficient mice [62]. In the brains of AD mouse models, plasmin and tPA activity are reduced, which could explain the progressive accumulation of parenchymal brain Aβ [62]. This finding is partially consistent with human data that show AD brains have low plasmin activity compared to brains from non-demented individuals [69]. However, other human studies concluded that plasmin levels/activity are unchanged in AD brains or cerebrospinal fluid (CSF) samples [70,71]. The inconsistency in plasmin activity in humans may be due to genetic factors, as a reduction in brain plasmin activity was found to correlate with the presence of the APOE epsilon 4 variant in AD patients [72]. However, additional studies are needed to confirm this hypothesis. On the other hand, silencing only blood-derived plasminogen using targeted antisense oligonucleotide technology reduces neuroinflammation and Aβ deposition in a mouse model of AD [73]. These data suggest that plasminogen may have different effects throughout the body: brain plasminogen appears to be beneficial as it may aid in Aβ clearance, whereas blood-derived plasminogen may be harmful as it can promote proinflammatory cascades that ultimately impair brain function. These findings highlight the importance of considering the location-specific effects of plasminogen in developing therapeutic strategies for AD.

### 4.2. Tissue Plasminogen Activator (tPA) 

In the vasculature, tPA is primarily expressed and secreted by endothelial cells located along the blood vessel walls. Here, tPA’s critical function is to convert plasminogen to plasmin, aiding in the regulation of blood clot formation and dissolution. In addition to its role in the blood, tPA has important functions in the brain, where it is synthesized and secreted by neurons and some glial cells. In the brain, tPA is involved in a variety of processes, including synaptic plasticity, neuronal survival, and neuroinflammation [74]. Overall, tPA is a critical protein involved in the regulation of both the vasculature and the central nervous system, and changes in its levels and activity are associated with different neurological disorders, including AD [67,74].

With regards to AD, it has been hypothesized by multiple labs that tPA directly participates in Aβ clearance. Studies have shown that tPA interacts with Aβ [75] and co-deposits alongside plasminogen with Aβ plaques in the Tg2576 AD mouse model [76]. In the hippocampus and amygdala of two AD mouse lines (TgCRND8 and Tg2576), tPA levels and activity are decreased, and injection of Aβ into the hippocampus of tPA-deficient AD mice leads to delayed Aβ clearance. These findings indicate that tPA may be involved in Aβ clearance in the brain, likely through the activation of plasmin [62,75]. Other groups have corroborated these findings in various other AD mouse lines [77,78,79,80,81]. For instance, treatment with recombinant tPA reduces Aβ burden in AD mice [79], and partial (heterozygous) or complete deficiency (homozygous) of tPA significantly worsens Aβ deposition and overall disease pathology in AD mice [77]. 

tPA may also be involved in CAA pathology in a mouse model of AD. However, the mechanism of how reduced tPA activity in the vasculature promotes CAA may be independent of plasmin and rather via NMDA (N-methyl-D-aspartate) receptor activation and nitric oxide release [80]. Interestingly, one study found that enhancing tPA activity by blocking PAI-1 (the main tPA inhibitor in the blood) did not reduce Aβ deposition in the brain but only affected CAA Aβ deposition [80]. This result suggests that increasing blood tPA activity reduced levels of Aβ40 but not Aβ42. In another study where brain slices were treated ex vivo, treatment with tPA in combination with plasminogen did not reduce the number of “mature” Aβ plaques, though it did reduce the amount and complexity of Aβ fibrils [76]. These data suggest that Aβ structure, fibril length, and aggregate complexity are important factors in the tPA-mediated clearance of Aβ in both blood and the brain. However, further research is needed to better understand tPA’s mechanistic role in the clearance of Aβ, especially with regard to different Aβ mutants and structural species.

Human data supporting the role of tPA in AD is complex and varied. Some studies report decreased tPA activity in AD patient brains or a negative correlation between tPA levels and Aβ load [81,82], which supports the role of tPA in Aβ clearance. However, another group reports an increase in total tPA protein in AD patients [83]. No changes in tPA levels in CSF or plasma have been reported in AD patients compared to non-demented control individuals [71,84]. However, the tPA/PAI-1 ratio is elevated in serum samples from MCI and AD patients, suggesting a reduction in tPA activity in their blood [85]. 

In addition to Aβ clearance, tPA has been linked to tau pathology in AD. A study using primary hippocampal neurons in culture suggests that tPA can cause tau phosphorylation through an ERK (extracellular signal-regulated kinase) signaling mechanism independent of plasmin [63]. Another group used primary neurons from the THY-Tau22 AD mouse model to show that tau affects the transport of tPA-containing vesicles in neurons [86]. However, further in vivo studies are necessary to better understand the role of tPA in tau pathology in AD.

Similar to tPA, uPA has been associated with plasmin-mediated clearance of Aβ in the brain [87]. It has also been suggested that uPA protects neurons from Aβ toxicity independently of plasmin by increasing the expression of cadherin [88]. The role of uPA in AD has been recently reviewed [88] and, therefore, will not be further discussed in the present review. 

### 4.3. Plasminogen Activator Inhibitor-1 (PAI-1) 

PAI-1 is a member of the serine protease inhibitor family. It plays a key role in the negative regulation of the fibrinolytic system by inhibiting the activity of tPA and uPA. PAI-1 also has been implicated in other functions, such as cell migration, angiogenesis, and wound healing [57,89]. Similar to plasminogen and tPA, PAI-1 is also reported to be involved in central nervous system physiology and pathology [90,91]. 

There is mounting evidence that suggests PAI-1 plays a role in the progression of AD. Elevated levels of PAI-1 have been observed in the brains of both AD mouse models and human patients, which correlates with an increase in Aβ load in both brain and the blood. For instance, in the APP/PS1 AD mouse line, PAI-1 levels increase over time as AD pathology worsens [90]. Moreover, PAI-1 deficiency in this mouse line then leads to reduced Aβ monomers, oligomers, and plaques in their brains [90]. Additionally, two independent studies using different PAI-1 inhibitors show a reduction in Aβ load and cognitive decline in AD mice [80,92]. Interestingly, these PAI-1 inhibitors targeted different types of Aβ, with TM5275 reducing both Aβ40 and Aβ42 load and PAI-039 only reducing Aβ40. The discrepancy in which Aβ form was reduced could be attributed to the use of different AD mouse models for these experiments, the nature of the PAI-1 inhibitors (i.e., different binding sites, efficacy, and off-target effects), or different timelines of PAI-1 inhibitor treatment.

The proposed mechanism by which PAI-1 inhibition leads to protection in AD is primarily associated with the increase in tPA and subsequent plasmin activity that results in the direct clearance of Aβ [80,90,92]. However, an increase in the maturation of BDNF after a decrease in PAI-1 inhibition may contribute to protection in AD [93], likely via an increase in plasmin and tPA activity. In contrast, an in vitro study using primary hippocampal neurons suggested that PAI-1 treatment may be neuroprotective in Aβ-mediated neurotoxicity [94]. However, to our knowledge, there are no in vivo studies further exploring this alternative hypothesis that PAI-1 could be neuroprotective in AD.

The findings from AD mouse model studies have led to increased interest in examining CSF or blood PAI-1 levels as a biomarker for AD in humans. One study found that PAI-1 plasma levels increase as AD progresses in humans [95]. Furthermore, in people with type 2 diabetes, a strong correlation was found between increased plasma PAI-1 levels in diabetics with MCI [96]. Another study found a positive correlation of PAI-1 levels with Aβ in the CSF of male but not female AD patients [97], indicating a potential sexual dimorphism of PAI-1 as an AD CSF biomarker. However, plasma PAI-1 levels were found to be increased in elderly, uneducated Chinese individuals with AD, but this finding was not observed in their educated AD counterparts [98]. These findings highlight the limitations of relying on a single biomarker to identify and diagnose AD, as disease populations are highly heterogeneous. 

In terms of genetic screenings, a polymorphism in the PAI-1 promoter (4G/4G) that increases PAI-1 transcription has been linked to an increased risk for AD [99]. However, this was not observed in the Chinese Han population [100] nor in a recent meta-analysis [101]. It is important to note that there is one study showing that PAI-1 levels are unchanged in the brains of AD patients, although they show reduced tPA activity [82]. In this small study, neuroserpin, another inhibitor of tPA in the brain, was increased instead of PAI-1 [82]. This result suggests that not only PAI-1 but other inhibitors of tPA and plasmin may be involved in AD pathology. Further research is necessary to elucidate the involvement of other inhibitors of these proteins in AD pathogenesis.

### 4.4. Fibrinogen 

Fibrinogen is a soluble plasma protein composed of three pairs of polypeptide chains—alpha, beta, and gamma—that is converted to an insoluble fibrin clot by the serine protease thrombin. Fibrinogen is secreted by platelets, hepatocytes, and endothelial cells in response to injury. After fibrinogen cleavage by thrombin, fibrin forms a mesh-like network that traps blood cells and other components to form a clot, preventing further bleeding and facilitating tissue repair. The cross-linking action of coagulation factor XIII then stabilizes the fibrin network. In several neurological disorders, including stroke and AD, fibrin(ogen) has been shown to extravasate into the brain [61,102]. There is substantial evidence showing that the fibrinogen beta chain binds to Aβ in vitro and in vivo, affecting clot formation, structure, and dissolution [103,104]. In AD mouse models, fibrin(ogen) deposits with Aβ in and around blood vessels, and its deposition in the brain parenchyma correlates with the progression of AD pathology, including neuronal death [46]. Numerous studies have shown that pharmacological or genetic reduction of fibrinogen reduces CAA pathology and/or parenchymal Aβ load and rescues behavioral deficits in various AD mouse lines [46,105,106,107]. The precise disruption of the Aβ/fibrinogen complex using the small molecule RU-505 leads to a reduction in CAA pathology, neuroinflammation, and cognitive decline in two AD mouse models [108], suggesting that fibrin(ogen) itself is not enough to induce AD pathology. Additionally, the interaction between fibrinogen and Aβ is proposed to enhance tPA-mediated plasminogen activation, which may contribute to CAA-associated intracerebral hemorrhage [33,109]. However, the Aβ/fibrinogen complex delays fibrin clot lysis [33]. This result suggests that the increase in plasmin activity may be ineffective in degrading Aβ/fibrinogen complexes and dissolving the clot, further contributing to CAA pathology. The molecular basis of how the fibrinolytic system directly participates in CAA and related pathologies requires further investigation.

Mechanistically, there is evidence that fibrinogen’s gamma chain is also involved in mediating AD pathology since targeting this region with a monoclonal antibody or an inhibitory peptide rescues neuropathology in AD mice [106,110]. This region is necessary for fibrinogen’s binding to the CD11b/Mac-1 (macrophage integrin) receptor, leading to microglial activation and promoting synaptic elimination in AD mice [111]. 

There is growing evidence linking fibrin(ogen) to AD pathology in humans. Increased fibrin(ogen) deposition has been observed in both blood vessels and the brain parenchyma of AD patients [105,111]. In addition, a recent study reported a positive correlation between plasma fibrinogen levels and Aβ and phosphorylated tau in the brains of AD patients [112]. However, two recent studies did not find a correlation between fibrinogen and AD risk or between brain deposition of fibrin(ogen) and Aβ or phosphorylated tau [113,114]. Therefore, further studies are needed to fully understand and clarify the role of fibrinogen in human AD pathology. Nonetheless, the use of plasma fibrinogen as an auxiliary biomarker in AD may be relevant for tailoring treatment strategies for individual patients.

**Figure 1:**
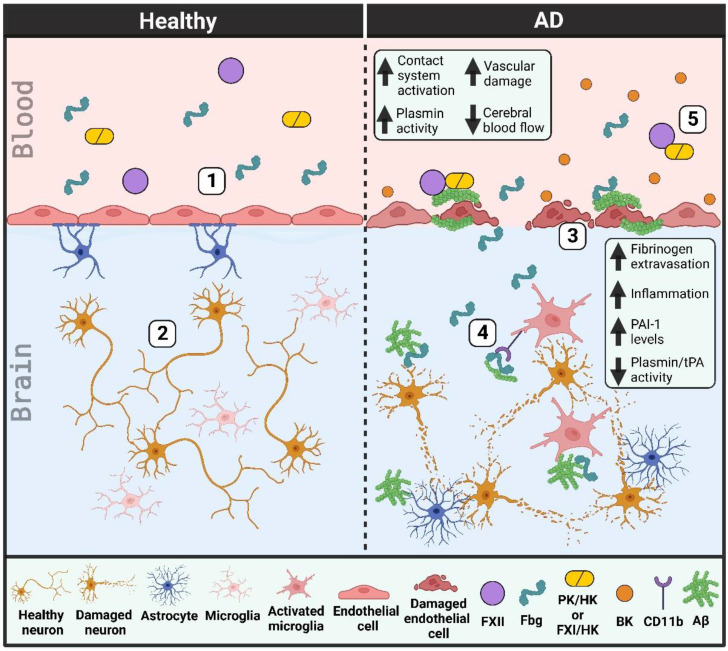
Vascular dysfunction in Alzheimer’s disease (AD) patient brains and blood vessels. (1) Healthy individuals have an intact blood-brain barrier. Endothelial cells are connected by tight junctions and lined by pericytes (not shown) and astrocytes. (2) This intact blood-brain barrier prevents the extravasation of blood proteins into the brain parenchyma, thereby helping to limit neuroinflammation and protect neurons from damage. (3) The blood-brain barrier in AD patients is damaged as tight junctions are lost and endothelial cells and pericytes (not depicted) degenerate. Cerebral blood flow is decreased due in part to an increase in beta-amyloid (Aβ) along blood vessel walls (cerebral amyloid angiopathy). (4) This vascular damage allows for the extravasation of blood proteins, like fibrinogen (Fbg), into the brain parenchyma. Fibrinogen may promote neuronal degeneration and induce an immune response in the brain, both through its interaction with Aβ and its binding to and activation of the CD11b/Mac-1 receptor on microglia. tPA and plasmin activities are decreased while PAI-1 levels are increased, limiting the removal of Aβ plaques from the brain parenchyma. (5) There is increased plasma contact system activation in AD patient blood due to enhanced interaction of plasma Aβ with factor XII (FXII). Bradykinin (BK) levels are increased, promoting neuroinflammation and further vascular damage. (PK, plasma kallikrein; FXI, factor XI; HK, high molecular weight kininogen).

A schematic that portrays vascular deficiencies involving the plasma contact system and the fibrinolytic system in AD can be found in Figure 1. 

## 5. Treatment Options for AD Focused on Vascular Deficiencies

Despite advances in research and medicine, treatment options for AD remain limited. Two recently FDA-approved therapeutics focus on targeting Aβ for removal from the brain. However, given the interplay between vascular dysfunction, coagulation, and inflammation in AD, many alternative therapies are appealing. These options include the potential of anticoagulant treatment, contact system inhibition, and small molecules or antibodies that can intercept toxic protein interactions in the bloodstream.

### 5.1. Anti-Amyloid Therapy

The two most recently approved therapies for AD, aducanumab and lecanemab, are anti-amyloid antibodies that reduce Aβ levels in the brain [18,19,20]. Both antibodies were found to provide modest but significant positive results in slowing cognitive decline in patients with AD. One major side effect of anti-amyloid treatment is amyloid-related imaging abnormalities (ARIA) [19]. Although some cases of ARIA are asymptomatic, some patients with ARIA experience edema, microhemorrhage, headache, dizziness, confusion, and/or nausea [19]. Individuals most affected by ARIA in these studies are those carrying an APOE epsilon 4 alleles. Of note, one lecanemab trial participant who was homozygous for APOE epsilon 4 experienced an ischemic stroke and was treated with recombinant tPA. The patient developed multiple intracerebral hemorrhages and later died [115]. Although recombinant tPA intervention in ischemic stroke patients may increase the risk of hemorrhagic transformation [116], the possible drug interaction between tPA and lecanemab treatment should be explored.

Clinical trials for anti-amyloid treatments have limited enrollment in patients with MCI or mild dementia [18,19]. The therapeutic potential of anti-amyloid treatment in patients who have late-stage AD/severe dementia remains to be determined. Similarly, the effect of anti-amyloid treatment on CAA has yet to be explored. Interestingly, as shown by many studies, the total Aβ burden in the brain does not always correlate with cognitive function in AD patients. However, it is known that the approved anti-amyloid treatments lower brain amyloid load, and both show relatively low penetration [117]. This raises the question as to how exactly these novel treatments are protective against disease progression. 

### 5.2. Anticoagulants

Patients and mouse models of AD show alterations in clotting and other hemostatic abnormalities. Studies in AD mice have found that chronic anticoagulant treatment is protective against inflammation [118] and cognitive dysfunction [119,120,121]. Anticoagulants inhibit clot formation and therefore reduce the proinflammatory effects of fibrin clots and the deposition of Aβ-containing clots. Numerous types of anticoagulants exist as vitamin K antagonists, direct or indirect thrombin inhibitors, FXa inhibitors, and unfractionated or low molecular weight heparins. They exhibit distinct mechanisms of action and target various critical proteins within the fibrinolytic cascade.

Vitamin K is a known pro-coagulant and serves as a cofactor for a number of clotting factors (factors II, VII, IX, and X), and its inhibition blocks coagulation. In addition, Vitamin K is a nutrient critical for the proliferation, differentiation, and survival of cells in the brain [122]. Thus, although the peripheral effects of vitamin K antagonists on coagulation may be protective, the effects on overall brain health may be deleterious. Treatment with Factor Xa inhibitors may bypass some of the negative effects of vitamin K antagonists by directly targeting another coagulation factor instead. 

Thrombin inhibitors act by preventing the formation of fibrin clots and thereby block the inflammatory processes driven by thrombin, fibrin, or Aβ/fibrin(ogen) complexes [123]. Clinically, dabigatran, a direct thrombin inhibitor, has been shown to have a significantly lower risk of stroke, intracranial hemorrhage, and even death compared to the Vitamin K antagonist, warfarin [124]. Dabigatran treatment can help rescue the reduced cerebral blood flow observed in AD patients and is thought to help preserve vascular integrity [121,123]. It is possible that anticoagulant treatment could be beneficial in AD. However, there are significant risks associated with anticoagulant treatment in elderly patients due to the increased risk of mortality or morbidity from intracerebral or gastrointestinal bleeding, which is further exacerbated by falls [125].

### 5.3. Contact System Inhibitors

The plasma contact system can promote coagulation and inflammation, both of which are pathologies observed in AD. Increased inflammation is due to the production of bradykinin and inflammatory nonapeptide that binds to bradykinin 1 and bradykinin 2 receptors throughout the body. Increased coagulation is due to the activation of the intrinsic clotting cascade via FXI. Inhibition of the plasma contact system in mice has been shown to have significant benefits in rescuing excessive inflammation and behavioral deficits in AD models [56]. Currently, there are no approved treatments for AD that inhibit the contact system. However, published studies have shown that both antibody-mediated [42,43] and antisense oligonucleotide-mediated inhibition [56] of contact system components could ameliorate disease pathology. The FDA has approved antisense oligonucleotide treatment for other diseases [126], raising the possibility for this type of therapy in AD as well. Antisense oligonucleotides have been particularly successful in specifically targeting proteins produced in the liver [126]. This option is promising as the proteins of interest in the plasma contact system and the fibrinolytic system are produced in the liver and secreted into circulation. The use of antibodies that inhibit the plasma contact system could prove beneficial as well. Administration of 3E8, a novel anti-HK antibody, has been shown to inhibit activation of the plasma contact system ex vivo and in vivo [42,43,127]. 3E8 targets HK and prevents the binding of PK and FXI to HK, thereby inhibiting both the inflammatory and thrombotic arms of the contact system [127]. Of note, inhibition of the intrinsic clotting pathway does not lead to bleeding complications [128], making this an attractive therapeutic approach.

### 5.4. Blocking Negative Effects and Interactions of Fibrinogen

Fibrin(ogen) is a key player in driving inflammation and neurodegeneration in disease. The interaction between Aβ and fibrin(ogen) alters clot structure, prevents clot lysis, and promotes neurodegeneration [32,105]. Reducing circulating fibrinogen levels [105], inhibiting the Aβ/fibrin(ogen) interaction [108,129], and inhibiting fibrin-mediated inflammation [110,111] have all been shown to have protective effects in AD models. The use of small molecules and/or antibodies to inhibit toxic protein interactions could be explored for novel therapies in AD. Inhibiting Aβ aggregation with the compound TDI-2760 prevents the Aβ/fibrin(ogen) interaction and limits Aβ-induced contact system activation [129]. Furthermore, inhibiting the Aβ/fibrin(ogen) interaction with RU-505 showed favorable effects in AD mouse models [108]: RU-505 restores normal clot lysis and reduces neurodegeneration in AD mice [108]. Similar molecules could have the potential to benefit AD patients. 

Alternatively, 5B8, a monoclonal antibody that binds to the gamma chain of fibrinogen, inhibits fibrin-induced inflammation and has shown protective effects in mouse models of AD [111]. Importantly, 5B8 does not interfere with normal clotting. 5B8 crosses the blood-brain barrier and binds to extravasated fibrinogen to prevent it from inducing an immune response via activation of the CD11b/Mac-1 receptor [111]. Collectively, the generation of a small molecule or antibody that blocks the Aβ/fibrin(ogen) interaction while also preventing extravasated fibrinogen from activating the CD11b/Mac-1 receptor would likely be beneficial in AD. Furthermore, certain familial mutations in Aβ have been linked to increased incidence of CAA and stronger binding of Aβ to fibrin(ogen) [33]. Patients with these mutations, which include the Dutch (E22Q Aβ) and Iowa (D23N Aβ) point mutations [33], would likely benefit from therapeutics focused on inhibiting Aβ/fibrin(ogen) binding.

The need for new diagnostic criteria that allows for early intervention is evident. The presence of vascular deficits early in AD and the detection of associated biomarkers may aid in identifying patients who are at increased risk for developing AD before cognitive decline begins. It is unlikely that one factor is solely responsible for driving disease pathology but rather the accumulation of numerous factors that accelerates degeneration and hence cognitive dysfunction. The identification of alternative pathways implicated in driving disease pathology suggests that combinatorial therapy may be beneficial in certain patients. It is possible that combining anti-amyloid treatments with contact system inhibitors, for example, could lessen Aβ load, mitigate inflammation, and prevent vascular damage before neuronal death and cognitive decline are severe. Table 1 presents the advantages and disadvantages of the therapies described in this review. 

## 6. Conclusions

This review provides compelling evidence for the involvement of the plasma contact and fibrinolytic systems in AD pathogenesis. The identification of possible mechanisms and therapeutic strategies involving these systems provides exciting avenues for future research. However, further studies are required to confirm the efficacy and safety of targeting the contact and fibrinolytic systems in AD patients. Importantly, combining treatment strategies targeting vascular damage, as well as Aβ pathologies, such as the combinatorial therapy of lecanemab to target Aβ protofibrils and a contact system inhibitor, could potentially lead to synergistic or additive effects to vastly improve patient outcomes. Overall, the identification of these novel treatment strategies targeting AD-related vascular damage is a promising opportunity for advancing AD patient care.

## Figures and Tables

**Table 1:** Advantages and disadvantages of novel therapeutics for addressing vascular deficiencies in AD.

Therapy	Advantages	Disadvantages
**Anti-Amyloid Antibodies** Lecanemab [18] Aducanemab [19,20]	Reduces Aβ load in the brain Improves cognition FDA approved	Intravenous administration Only tested in mild AD patients Mechanism of action not fully understood Complications of ARIA
**Contact System Inhibitors** 3E8 anti-HK antibody [42,43,127] Antisense oligonucleotide technology [56,126]	Reduces inflammation Improves cognition Does not interfere with normal clotting Multiple protein targets available (FXII, FXI, PK, HK)	Not FDA approved yet Testing needed in humans
**Fibrinogen Blockers** 5B8 anti-fibrinogen antibody [110] Small molecules RU-505 [108] and TDI-2760 [129] that inhibit Aβ/fibrinogen complex formation	Limits inflammation Does not interfere with clotting Multiple protein targets available (Aβ, fibrinogen, and/or CD11b)	Not FDA approved yet Testing needed in humans
**Anticoagulants** [121,125] Heparin Vitamin K antagonist Factor Xa inhibitor	Reduces inflammation Improves cognition Reduces risk of stroke	May promote internal bleeding in elderly patients

## Data Availability

Not applicable.
